# A Proprietary *Punica granatum pericarp* Extract, Its Antioxidant Properties Using Multi-Radical Assays and Protection Against UVA-Induced Damages in a Reconstructed Human Skin Model

**DOI:** 10.3390/antiox14030301

**Published:** 2025-02-28

**Authors:** Steve Thomas Pannakal, Steven Durand, Julie Gizard, Peggy Sextius, Emilie Planel, Emilie Warrick, Damien Lelievre, Celine Lelievre, Joan Eilstein, Floriane Beaumard, Arpita Prasad, Sanketh Shetty, Arun Duraisamy, Kumar Gaurav, Sherluck John, Adrien Benazzouz, Xavier Fastinger, Dhimoy Roy, Vishal Sharma

**Affiliations:** 1 L’Oréal Research and Innovation, Bangalore 560067, India; 2 Episkin, 4 Rue Alexander Fleming, 69007 Lyon, France; 3 L’Oréal Research and Innovation, 93600 Aulnay Sous-Bois, France; 4 L’Oréal Research and Innovation, Mumbai 400043, India

**Keywords:** *Punica granatum*, POMAOX, polyphenols, ellagic acid, punicalagins, antioxidant, fibroblasts, T-Skin^TM^, UVA, photoprotection

## Abstract

**Background:** Within the solar ultraviolet (UV) spectrum, ultraviolet A rays (UVA, 320–400 nm), although less energetic than ultraviolet B rays (UVB, 280–320 nm), constitute at least 95% of solar UV radiation that penetrates deep into the skin The UV rays are associated with both epidermal and dermal damage resulting from the generation of reactive oxygen species (ROS). Among them, the longest UVA wavelengths (UVA1, 340–400 nm) can represent up to 75% of the total UV energy. Therefore, UVA radiation is linked to various acute and chronic conditions, including increased skin pigmentation and photoaging. Despite many advances in the skin photoprotection category, there is still a growing demand for natural daily photoprotection active ingredients that offer broad protection against skin damage caused by UVA exposure. In our quest to discover new, disruptive, next generation of photoprotective ingredients, we were drawn to pomegranate, based on its diverse polyphenolic profile. We investigated the pericarp of the fruit, so far considered as byproducts of the pomegranate supply chain, to design a novel patented extract “POMAOX” with a desired spectrum of phenolic components comprising of *αβ*-punicalagins, *αβ*-punicalins and ellagic acid. **Methods**: Antioxidant properties of POMAOX were measured using in-tubo standard tests capable of revealing a battery of radical oxygen species (ROS): peroxyl radical (ORAC), singlet oxygen (SOAC), superoxide anion (SORAC), peroxynitrite (NORAC), and hydroxyl radical (HORAC). In vitro, confirmation of antioxidant properties was first performed by evaluating protection against UVA-induced lipid peroxidation in human dermal fibroblasts (HDF), via the release of 8 iso-prostanes. The protection offered by POMAOX was further validated in a 3D in vitro reconstructed T-Skin^TM^ model, by analyzing tissue viability/morphology and measuring the release of Matrix Metallopeptidase 1 (MMP-1) & pro-inflammatory mediators (IL-1α, IL-1ra, IL-6, IL-8, GM-CSF, and TNF-α) after UVA1 exposure. **Results:** POMAOX displayed strong antioxidant activity against peroxynitrite (NORAC) at 1.0–3.0 ppm, comparable to the reference vitaminC, as well as singlet oxygen (SOAC) at 220 ppm, and superoxide radicals with a SORAC value of 500 ppm. Additionally, POMAOX demonstrated strong photoprotection benefit at 0.001% concentration, offering up to 74% protection against UVA-induced lipid peroxidation on HDF, in a similar range as the positive reference, Vitamin E at 0.002% (50 µM), and with higher efficacy than ellagic acid alone at 5 µM. Moreover, our pomegranate-derived extract delivered photoprotection at 0.001%, mitigating dermal damages induced by UVA1, through inhibition of MMP-1 and significant inhibition of pro-inflammatory mediators release (including IL-1α, IL-1ra, IL-6, IL-8, GM-CSF, and TNFα) on an in vitro reconstructed full-thickness human skin model with a similar level of protection to that of Vitamin C tested at 0.035% (200 µM). **Conclusions**: Overall, the novel pomegranate-derived extract “POMAOX” significantly reduced the impact of UVA on human skin, due to its broad-spectrum antioxidant profile. These findings suggest that POMAOX could offer enhanced protection against the detrimental effects of UV exposure, addressing the growing consumer demand for strong photoprotection with skincare benefits.

## 1. Introduction

Solar radiation mainly comprises wavelengths ranging from 200 to 4000 nm of which, around 50–55% is infrared radiation (IR, 800–2500 nm), 40–45% is visible light (VIS, 400–800 nm), and 5% ultraviolet radiation (UV, 200–400 nm) [1,2]. UVA rays (320–400 nm) amount to over 90% of the total UV radiation reaching the earth’s surface. UVA radiation is further subdivided into longwave UVA1 (340–400 nm) and shortwave UVA2 (320–340 nm). UVA photons penetrate deep into the epidermis and dermis of the skin [3]. About 80% of UVA radiation reaches the dermo-epidermal junction and penetrates into the papillary dermis, while, both UVA and UVB contribute to reactive oxygen species (ROS) generation and oxidative stress [4], UVA penetrates deeper into the skin leading to the production of ROS into the dermis [5]. These ROS, including superoxide anion radicals (O_2_^•−^), hydroxyl radicals (•OH), and their active precursors, namely, singlet oxygen (^1^O_2_) and hydrogen peroxide (H_2_O_2_), cause not only protein oxidative damage but also lipid peroxidation. The production of peroxyl radicals damages cell membranes and mediates the secretion of various inflammatory cytokines as well as matrix metalloproteinases (MMPs), which accelerate the breakdown of dermal collagen fibers and extracellular matrix (ECM), increasing skin wrinkles. Consequently, the cell membrane fluidity is substantially decreased while, the UVA exposure also triggers depolarization of the mitochondrial membrane, which compromises the formation of adenosine triphosphate (ATP) and disrupts the energy homeostasis, leading to extensive cellular damage or cell death [6].

Naturally occurring antioxidants have gained considerable attention for their potential to prevent UV radiation-induced oxidative damage, directly via reacting with free radicals or indirectly by inhibiting the activity or expression of free radical-generating enzymes or enhancing the activity or expression of intracellular antioxidant enzymes. Polyphenols are the most abundant antioxidants as part of the human diet and a major part of antioxidants are found in pomegranates, oranges, grapes, and apples. Pomegranate also known as grenade, granats, and *Punica* apple, is a fruit of the *Punica granatum* L. tree from the family Lythraceae. It is indigenous to the Himalayas in northern India and distributed throughout Iran, parts of Southeast Asia, the East Indies, and tropical Africa, and grows in almost all parts of the Mediterranean region [7,8,9]. Pomegranate is rich in tannins including ellagitannins and gallotannins, phenolic acids, flavanols, flavones, flavanones, anthocyanidins, and anthocyanins. Over the last decade, the pomegranate fruit extract has been extensively studied for its antioxidant and anti-inflammatory properties. A few in vitro and in vivo studies have been reported with the photoprotective benefit of pomegranate against UVB-induced radiation [10], as well as against UVA-induced MMP-1 activity in normal human primary dermal fibroblast-neonatal (HDF-N) cells, compared to that of UVA-exposed cells [11]. Additionally, pomegranate fruit extract treated on normal human epidermal keratinocytes (NHEK) resulted in a dose-dependent inhibition of the UVA-mediated phosphorylation of signal transducer and activator of transcription 3 at Tyr705, AKT at Ser473, and ERK1/2, as it offers antioxidant protection against the carcinogenic effects of excessive sun exposure through activation of AKT on UV irradiation [12]. Clementi et al. in 2020, reported the protective effects of *αβ*-punicalagins on reducing high levels of ROS associated with UVA-induced oxidative stress through Nrf2 activation, thereby promoting Nrf2 nuclear translocation and upregulating its downstream antioxidant target genes (HO-1 and NQO1) as well as demonstrating an anti-apoptotic effect by decreasing Bax/Bcl-2 ratio [13]. Although the cosmetic benefits of pomegranate are well documented, no comprehensive study has been undertaken on the protective properties of pomegranate pericarp extract against UVA1-induced dermal damages using a reconstructed full-thickness human skin model and comparing the efficacy to that of Vitamin C, a cosmetic reference widely used today.

Despite the many advances in the photoprotection skin category, there is still a growing demand for natural daily photoprotection active ingredients that offer broader protection against skin damage caused by UVA exposure. Additionally, it is well known that some UV filters do not protect the skin efficiently against the full UV spectrum, especially in the UVA1 domain, and therefore do not fully prevent ROS generation and its consequences. Therefore, the addition of antioxidants in photoprotective formulations can offer new ways to prevent oxidative damage caused by ROS [14]. In our quest to discover new, disruptive photoprotective ingredients, we were drawn to the non-edible part of the pomegranate pericarp, which is generally regarded as waste and disposed of as compost or for the generation of biogas. Interestingly, the pericarp has a diverse polyphenolic profile that drew our attention to engineer a novel patented extract “POMAOX” using a one-pot green extraction with a desired spectrum of phenolic components comprising of <42% αβ-punicalagins, αβ-punicalins, and ellagic acid. POMAOX is readily biodegradable and ecofriendly by design and our one-pot green extraction optimized process enabled the sustainable exploitation of waste and byproducts of the pomegranate supply chain. Additionally, the process was innovative and offered a commercially viable approach to deliver a specific polyphenol composition with high efficacy and remains relevant in the context of a cosmetic industrial environment.

## 2. Materials and Methods

### 2.1. Raw Material, Solvents, Chemicals and Standards

The pericarp of *Punica granatum* was collected from the Ramban district of the Indian state of Jammu and Kashmir, (kanga village) during the period of September to December, more particularly at the latitude and longitude around 32° N, 74° E, and the specimen was authenticated by a taxonomist. Voucher specimens of the pericarp have also been deposited at the Herbarium facility at L’Oreal (Advanced Research, Bangalore, India), with voucher specimen numbers MA/L’Oreal/R&D-0026/27/28/29. They were stored under frozen conditions at −20 °C and protected from light. A series of tests were conducted on the fresh pericarp, while a separate fraction was subjected to drying at 45 °C for a duration of 12 h in a ventilated laboratory oven (FALC Instruments, Treviglio, Italy). In order to determine the moisture content and proportions of organic and inorganic fractions, a thermogravimetric investigation was carried out using a muffle furnace (Nabertherm GmbH, Lilienthal, Germany). The experimental protocol involved dehydration at 100 °C for 12 h, followed by calcination at 650 °C for 4 h (see Table 1). All solvents and reagents utilized in this study were purchased from Sigma-Aldrich (St. Louis, MO, USA). HPLC–MS-grade acetonitrile and methanol were purchased from Merck (Lowe, NJ, USA). Laboratory-grade ethanol was obtained from Merck Millipore (Darmstadt, Germany). Milli-Q Integral 15 system (Merck Millipore, Burlington, MA, USA) was used to prepare the HPLC-grade water. High purity analytical standards of ellagic acid, punicalins (A + B mixture), and punicalagins (A + B mixture) were obtained from Sigma–Aldrich Chemical Co. (St Louis, MO, USA). AAPH was obtained from Cayman Chemical Co. (Ann Arbor, MI, USA; product No. 82215). Fluorescein salt solution (FL; product No. 46960), (±)-6-hydroxy-2,5,7,8-tetramethylchroman-2-caroboxylic acid (Trolox; product No. 238813), and phosphate-buffered saline (PBS) tablets were obtained from Sigma Aldrich (St. Louis, MO, USA). DPBF was obtained from Tokyo Kasei Organic Chemicals, Japan, and endoperoxide (EP) was obtained from Wakenyaku Co. Ltd., Tokyo, Japan.

### 2.2. Extraction Punica granatum pericarp Using One Pot Green Extraction Process

**Figure 1:**
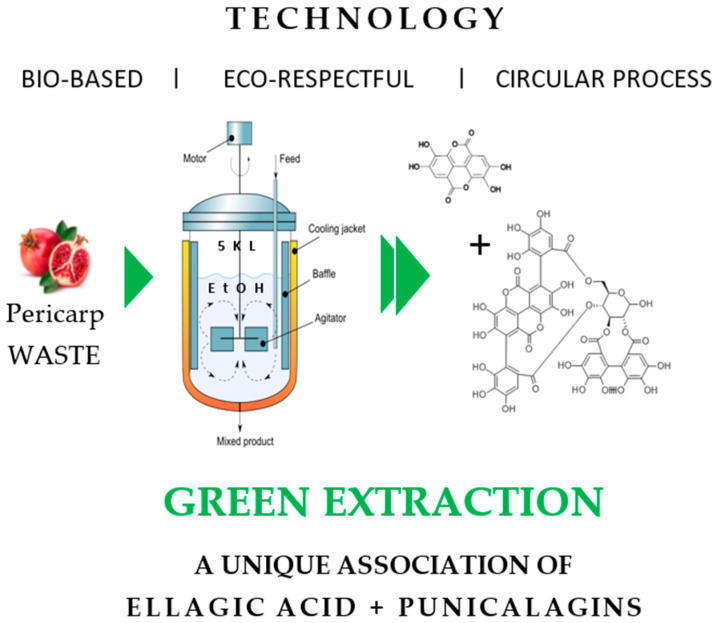
Green Extraction Technology of POMAOX.

The dried pericarp of *Punica granatum* (1 kg) was extracted at 42 °C with 5 L of ethanol (Proof 99.5%) with stirring at 800 rpm for a brief period of 4 h. The ethanolic extract obtained was 5 filtered through a Buchner funnel containing cotton cloth (mesh size: 20 microns). The filtered extract was evaporated under vacuum at 38 °C, to afford a brown amorphous powder. The extraction is repeated successively for two consecutive times under the conditions explained above (Figure 1). The combined dried extract consists of the major phenolic compounds i.e., ellagic acid, punicalins, and punicalagin. The extract was subjected to stability studies under different thermal conditions (4 °C, rt, and 45 °C) for a duration of 2 months. The extract was found to be stable with a total polyphenol stability >80%.

### 2.3. In Tubo Antioxidant Assays

#### 2.3.1. Oxygen Radical Absorbance Capacity (ORAC)

The ORAC assay was performed following the protocol of Ou and co-workers (2013) with modifications [15]. In a 96-well black plate (Costar, Corning Incorporated, Glendale, AZ, USA), 0.150 mL of 111.2 nM Fluorescein working solution was mixed with 0.025 mL of either Trolox standard solution, sample solution, or ORAC buffer (75 mM phosphate buffer pH 7.2) as a blank solution. After a 30-min incubation at 37 °C, 0.025 mL of 153 mM 2,2′-azobis (2-amidinopropane) dihydrochloride (AAPH) solution, a free radical generator, was added to the reaction solution. Fluorescence was measured every 1 min for 1.5 h at an excitation wavelength of 485 nm and emission wavelength of 535 nm (Ex485/Em535) at a constant temperature of 37 °C. The antioxidant activity was measured by calculating the area under the fluorescence curve (AUC), and then comparing the AUC of the sample to that of the control (without antioxidant). Trolox was used as a positive control for the assay.

#### 2.3.2. Superoxide (O_2_^•–^) Anion Scavenging Assay (SORAC)

SORAC assay was carried out following the methods of Zhang and co-workers (2009) [16] with modifications. A substrate-sample solution comprising 0.1 mL of 3.2 µM HE in 1.0 mM xanthine solution and 0.050 mL of sample solution was incubated for 20 min at 37 °C were mixed together in a 96-well black plate. Xanthine oxidase solution (0.050 mL) was added to initiate the reaction, and fluorescence was measured every 2 min for 0.5 h at an excitation wavelength of 485 nm and emission wavelength of 590 nm (Ex485/Em590). Trolox was used as a positive control for the SORAC assay, whereas ORAC buffer (75 mM phosphate buffer pH 7.2) was used as a blank solution.

#### 2.3.3. Hydroxyl (HO•) Radical Scavenging Capacity (HORAC)

The HORAC assay was performed according to the protocol of Ou and co-workers (2002) [17] with modifications. In a 96-well black plate, 0.1 mL of 0.124 µM Fluorescein solution was mixed with 0.050 mL of sample solution and 0.025 mL of 0.80% hydrogen peroxide. After incubation at 37 °C for 5 min, 0.025 mL of cobalt solution (24 mg cobalt chloride and 30 mg picolinic acid per 10 mL) was added, and fluorescence was measured every minute for 1.5 h at an excitation wavelength of 485 nm, and an emission wavelength of 520 nm (Ex485/Em520). Gallic acid was used as a positive control for the HORAC assay, with ORAC buffer (75 mM phosphate buffer pH 7.2) used as a blank solution.

#### 2.3.4. Singlet Oxygen (^1^O_2_) Scavenging Capacity (SOAC)

The SOAC assay was done in accordance with the methods of Ou and co-workers (2012) with modifications [18,19,20]. In a 96-well black plate, 0.115 mL of 40 µM Endoperoxide (EP) in 1,3-Diphenylisobenzofuran (DPBF) solution was mixed with 0.025 mL of sample solution. After incubation at 37 °C for 20 min, the following reagents were added sequentially: 0.025 mL of 8.4 mM Na_2_MoO_4_, 0.025 mL of 0.16% hydrogen peroxide solution, and 0.010 mL of 0.1 M NaOH. Fluorescence was measured every 2 min for 1.0 h at an excitation wavelength of 485 nm and an emission wavelength of 590 nm (Ex485/Em590). Tocopherol was used as a positive control for the SOAC assay, whereas ORAC buffer (75 mM phosphate buffer pH 7.2) was used as a blank solution.

### 2.4. Lipid Peroxidation Assay on Human Dermal Fibroblasts

#### 2.4.1. Cell Culture

Human dermal fibroblasts (HDFs) isolated from neonatal foreskin were purchased from Thermo Fischer Scientific (Waltham, MA, USA). Cells were cultured in DMEM supplemented with 10% SVF at 37 °C, 5% CO_2_.

#### 2.4.2. Cell Viability Assay

HDFs were treated for 24 h with POMAOX at 4 concentrations before being exposed to UVA (20 to 25 J/cm^2^) using a 1600 W Xenon lamp equipped with a dichroic mirror (Oriel, les Ulis, France) + WG335 filter (Schott, Clichy, France). Cell viability was assessed using a Calcein-AM probe (Molecular Probes, Thermofisher Scientific, Villebon-sur-Yvette, France). Briefly, 24 h after exposure, the cells were incubated with the probe diluted to 1/1000 in the culture medium for 30 min at 37 °C, 5% CO_2_. After rinsing out the medium with phosphate-buffered saline (PBS), cells were lysed with 200 µg/mL SDS and fluorescence was read with a spectrofluorimeter (SpectraMax, Molecular Devices, San Jose, CA, USA).

#### 2.4.3. 8-Isoprostane Release by Human Dermal Fibroblasts

HDFs were seeded in 48-well plates and treated with POMAOX or the reference Vitamin E (alpha-tocopherol) for 24 h at 37 °C, 5% CO_2_. After the treatment, cells were rinsed and exposed to UVA in PBS, before incubation for 2 h 30 min at 37 °C, 5% CO_2_. The amount of secreted 8-isoprostane in the supernatant of UVA- or sham-exposed cells was measured using an enzyme immunoassay (Cayman Chemical, Ann Arbor, MI, USA), according to the manufacturer’s instructions.

### 2.5. Protection Against UVA1-Induced Damage on Reconstructed Full Thickness T-Skin^TM^ Model

1. In vitro skin model: T-Skin^TM^ tissue model production

The T-Skin^TM^ model (Episkin, Lyon, France) was prepared in accordance with the method provided by D. Lelièvre et al. [21]. This skin model was made of a fibroblast-populated dermal equivalent (a mixture of collagen type I) on which normal human adult primary keratinocytes were seeded [22]. The keratinocyte and fibroblast strains were isolated from breast and abdominal normal skin samples respectively, obtained from surgical residues after written informed consent from the donors. The culture was left for 11 days in submerged conditions and then lifted at the air-liquid interface for 7 days to obtain a fully differentiated epidermis.

2. Preliminary MTT assay

Tissue viability was measured on a first T-Skin^TM^ tissue batch in duplicate (2 inserts per condition) using the MTT Reduction Assay. Briefly, tissues were transferred in 2 mL of MTT at 1 mg/mL in 6-well plates for 3 h at 37 °C, 5% CO_2_. A punch of 0.8 mm was used to remove a reproducible tissue surface for each sample. The epidermis was separated from the dermis and after overnight extraction in isopropanol, the concentration of the viable cells was measured using Optical Density (OD) at 570 nm. The mean OD of no treated control tissues non-exposed to UVA1 corresponded to 100% of tissue viability. Viability percentages of the other conditions were normalized by this control.

Active concentrations were considered non-cytotoxic if the viability percentage was higher than 65% in both cutaneous compartments.

3. Long UVA photoprotection test on T-Skin^TM^ model:

- Treatment:

Three batches of T-Skin^TM^ were treated systemically with POMAOX (0.0001 & 0.001% in 0.1% DMSO) and with Vitamin C at 200 µM in duplicate for 24 h. After 24 h incubation, tissues were exposed to UVA1 at 35 J/cm^2^ with Oriel 6″ × 6″ solar simulators equipped with WG360 0.8 mm filter (340 nm to 400 nm) which corresponded to the Biological Efficient Dose (BED) in this model, defined as the minimal dose causing fibroblast disappearance with no to mild epidermal alteration [23]. After UVA1 exposure, tissues were treated for 48 h the same way as in preventive treatment. Tissues were then cut and fixed in a formalin solution (4% *w*/*v*) before being dehydrated and embedded in paraffin. The culture media were collected and stored at −80 °C for soluble mediators quantification.

- Histology analysis and fibroblast counting:

For each condition, 3 sections (5 µm thickness) spaced out of 100 µm were cut per paraffin block. These slides were stained with Hematoxylin-Eosin-Saffron (HES) on a Sakura robot and scanned using the Nanozoomer (NDPscan software 2.5.89 version) to obtain virtual slides with 20× resolution and brightfield scanning profile.

For the histology analysis, 2 representative fields of each tissue and each slide which correspond to 6 fields per tissue and 12 fields per condition & per T-SkinTM batches were extracted using the NDPview software (version 1.2.55) with X20 HES Field.

Fibroblast counting was done manually on these 12 fields.

- MMP-1 and cytokines dosing:

#### 2.5.1. MMP-1 Protein Release

Matrix metalloproteinase 1 (MMP-1) was quantified in the culture media using Quantikine MMP-1 R&D (SMP100) (Biotechne^®^, Minneapolis, MN, USA) which was used according to the supplier’s instructions and the laboratory practices. The 96-well plate was read on the spectramax M5E and the results were analyzed using the SoftmaxPro software, version 7.1 GxP. The protein amounts (in ng/mL) were calculated using the sample dilution factor (1/250) and normalized with the mean fibroblast number of the corresponding condition.

#### 2.5.2. Pro-Inflammatory Mediators Quantification (IL-1a, IL-1ra, IL-6, IL-8, GM-CSF, and TNF-α) Using a Bio-Plex200

Proinflammatory cytokines were quantified in the culture media using a Bio-Plex 200, Luminex multiplex protein assay (Biorad, Marnes-la-Coquette, France). The kit allows the measurement of Interleukin (IL)-1α, IL-1 receptor antagonist (IL-1ra), Tumor Necrosis Factor (TNF)-α, IL-6, IL-8 and Granulocyte-Macrophage Colony-Stimulating Factor (GM-CSF) which were quantified using dilution factor 1/50 for IL-6 & IL-8 and a factor of 1 for IL-1ra, IL-1α, GM-CSF and TNF-α. Results were analyzed using the Bio-Plex Manager software, version 6.2 with a 5PL analysis. Protein amounts (in pg/mL) were calculated taking into account the sample dilution factor and normalized with the mean fibroblast number of the corresponding condition.

For POMAOX and Vitamin C, the interpretation of the photoprotective activity was established in comparison to the UVA-exposed vehicle control, respectively DMSO 0.1% and culture medium (untreated, UVA1-exposed condition).

## 3. Results

### 3.1. In Vitro Antioxidant Assays

The total polyphenol content of POMAOX was determined using the Folin-Ciocalteu assay. The method was applied to quantify total polyphenolic content in POMAOX and found to be 42% *w*/*w*. This novel patented extract was evaluated for its antioxidant activity against a battery of radical species: peroxyl (ORAC), singlet oxygen (SOAC), superoxide anion (SORAC), peroxynitrite (NORAC), and hydroxyl (HORAC) using standard protocols. The results for the five assays are summarized in Table 2. POMAOX did not show antioxidant activity against oxygen radicals (ORAC), but displayed strong antioxidant activity against singlet oxygen (SOAC) at 220 ppm and antioxidant activity against superoxide radicals with a SORAC value of 500 ppm against peroxynitrite (NORAC) and followed the same trend observed with SORAC, having the highest value at 1.0–3.0 ppm compared to the reference, vitamin C respectively. These findings support the strong antioxidant properties of POMAOX.

### 3.2. Protection Against UVA-Induced Lipid Peroxidation in Human Dermal Fibroblasts

**Figure 2:**
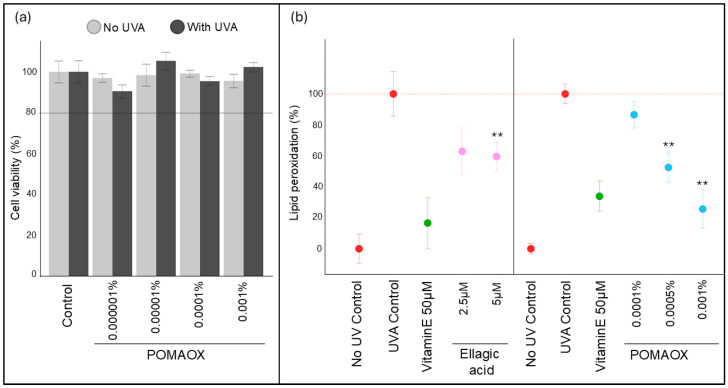
Evaluation of the photoprotective potential of POMAOX on lipid peroxidation under UVA exposure in human dermal fibroblasts (HDFs). (**a**) Evaluation of POMAOX cytotoxicity in UVA- or sham-exposed (No UVA) HDFs, (**b**) UVA-induced lipid peroxidation in HDFs pre-treated for 24 h with POMAOX or pure ellagic acid. Vitamin E (alpha-tocopherol) at 50 µM was used as a positive reference. The release of 8-isoProstanes (8isoP) in the cell supernatants was assayed 2 h 30 after UVA exposure. Lipid peroxidation is expressed as a percentage of 8isoP release by treated, UVA-exposed cells compared to untreated, UVA-exposed cells (UVA control, 100%). The release of 8isoP in untreated, sham-exposed cells (No UV control) is used as a baseline. Bar graphs represent mean ± SD of 3 independent experiments. ** Significant difference compared to the UV-exposed control (*p* < 0.01, Mann Whitney statistical test).

A preliminary cytotoxicity assessment of POMAOX on human dermal fibroblasts (HDFs) was performed using the calcein assay. UVA exposure induced a non-significant 13% decrease in cell viability compared to sham-exposed control. POMAOX was non cytotoxic at all concentrations tested, either with or without UVA exposure (Figure 2a). HDFs were then treated either with the POMAOX extract at 0.0001%, 0.0005% 0.001%, or with pure ellagic acid at 2.5 µM (0.00008%) and 5 µM (0.00015%) for 24 h and were exposed to UVA. 8-isoprostanes (8isoP) release as a marker of lipid peroxidation was assayed 2.5 h after UVA exposure. Untreated, sham-exposed cells (No UV control) and untreated, UVA-exposed cells (UVA control) were used as controls. The amount of 8isoP in untreated, sham-exposed cells (No UV control) was used as the baseline and the induction of 8isoP in untreated, UVA-exposed cells (UVA control) was set to 100%. The release of 8isoP by HDFs pre-treated with POMAOX at 0.0001%, 0.0005%, and 0.001% was 87%, 52%, and 26% respectively, representing protection against lipid peroxidation of 13%, 48% and 74% compared to the UVA control (Figure 2b). Vitamin E (50µM, 0.002%) afforded 64% protection against lipid peroxidation. POMAOX at 0.001% thus displayed a similar level of protection as Vitamin E at 0.002%. In HDFs pre-treated with Ellagic acid at 2.5 µM (0.00008%) and 5 µM (0.00015%), the percentage of lipid peroxidation was decreased by 37% and 40% respectively compared to the UVA control, which was lower than the protection offered by Vitamin E. Therefore, the POMAOX extract at 0.001%, a concentration corresponding to an approximate content of 3.3 µM ellagic acid, displayed better protection against lipid peroxidation than pure ellagic acid alone at a similar concentration.

### 3.3. Protection Against UVA1-Induced Damage on Reconstructed T-Skin^TM^ Model

- Preliminary MTT test

Epidermal and dermal viability values were measured 48 h after UVA1 exposure at a dose of 35 J/cm^2^ in T-Skin™ samples, pre-treated or not with POMAOX. MTT results did not show significant changes in the viability percentage after treatment with POMAOX at 0.0001 and 0.001% but a decrease occurred at 0.005%.

POMAOX was then determined to be non-cytotoxic at concentrations 0.0001 and 0.001% but to have deleterious impacts on the tissue after UVA1 exposure for concentrations above 0.005% (Table 2).

- Histology analysis

**Figure 3:**
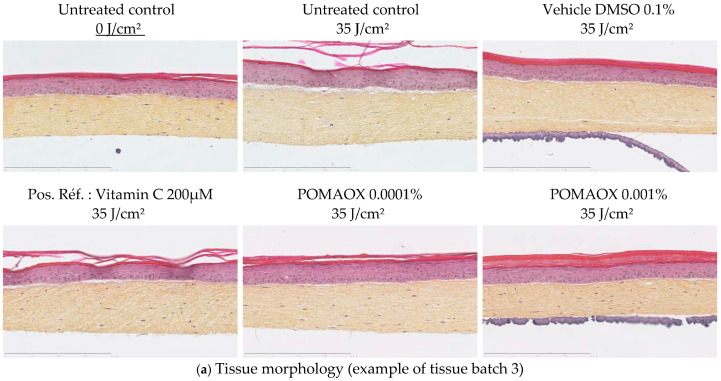

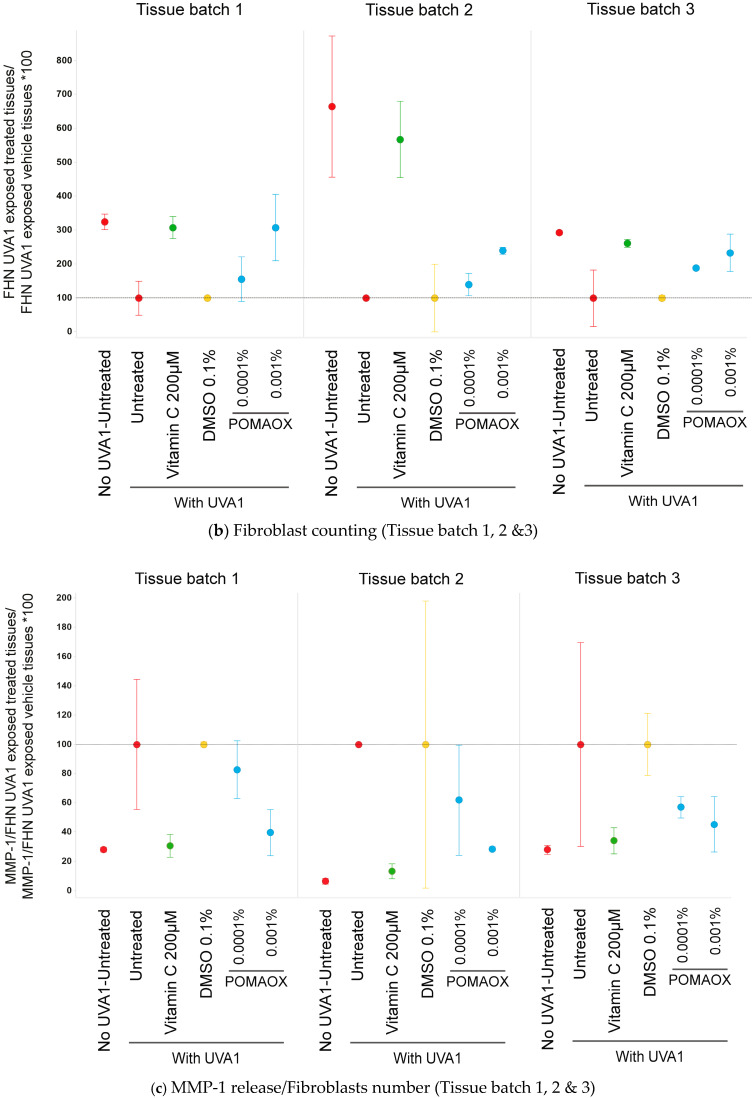

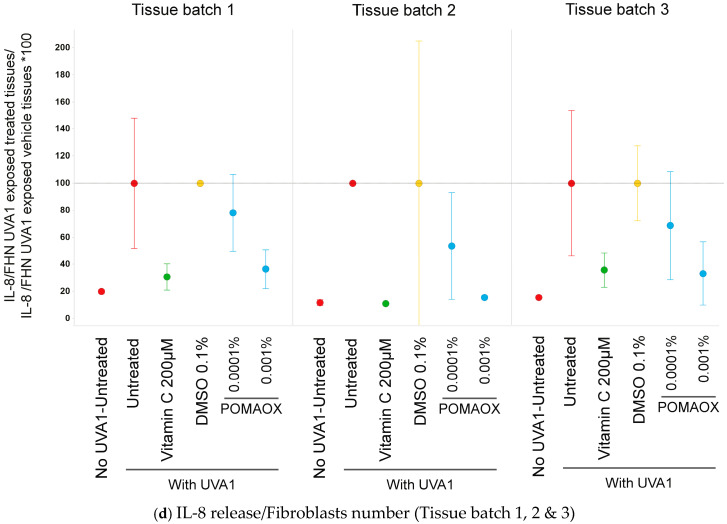
Evaluation of the photoprotective potential of POMAOX against UVA1-induced damage on reconstructed T-Skin^TM^ model. All the UVA1 exposures were done at 35 J/cm^2^. Vitamin C at 200 µM was used as a positive reference. MMP-1 and cytokines release were normalized for each condition by corresponding fibroblasts number. (**a**) Tissue morphology (HES histology) in sham-exposed (No UVA1), untreated UVA1-exposed, vehicle exposed, VitC 200 µM exposed, POMAOX 0.0001% and 0.001% exposed tissues (**b**) Dermal fibroblasts counting on tissues pre-treated for 24 h with POMAOX, then UVA1 exposed and post-treated for 48 h. Fibroblast number is expressed as a percentage of fibroblast number in POMAOX or Vitamin C treated and exposed compared to exposed vehicle control respectively DMSO 0.1% and culture medium (untreated). Scatter plot represents mean ± SD of 3 independent experiments. (**c**) The release of MMP-1 in the supernatants was assayed 48 h after UVA1 exposure. MMP-1 release is expressed as a percentage of MMP-1 release in POMAOX or Vitamin C treated and exposed compared to exposed vehicle control respectively DMSO 0.1% and culture medium (untreated). Scatter plot represents mean ± SD of 3 independent experiments. (**d**) IL-8 quantification in the supernatants was assayed 48 h after UVA1 exposure. Il-8 level is expressed as a percentage of IL-8 level in POMAOX or Vitamin C treated and exposed compared to exposed vehicle control respectively DMSO 0.1% and culture medium (untreated). Scatter plot represents mean ± SD of 3 independent experiments.

The untreated tissues exposed at 35 J/cm^2^ were impacted with slight alterations of the epidermis and disappearance of fibroblasts (Figure 3a), as previously reported [24].

Treatment with POMAOX led to an improvement of the morphology of the whole reconstructed skin, a decrease in morphological disorganization of the tissue, and mainly to a decrease of fibroblasts disappearance (Figure 3a) with a visible dose effect, which was confirmed by quantification of the density of fibroblasts in the dermis (Figure 3b).

- MMP-1 and cytokines quantification

After UVA1 exposure at 35 J/cm^2^, the release of MMP-1 and pro-inflammatory mediators levels (IL-1α, IL-1ra, IL-6, IL-8, GM-CSF, and TNF-α) in the culture medium of reconstructed skin samples was quantified and normalized by dermal fibroblast number. After UVA1 exposure all the protein levels were comparatively higher than levels measured in unexposed samples (0 J/cm^2^). The level of MMP-1 normalized by dermal fibroblast number is presented in Figure 3c. In samples treated with POMAOX at 0.001%, there was a decrease in MMP-1 production comparable to the effect obtained after treatment with Vitamin C, indicating a strong photoprotective effect. Our results show that POMAOX can prevent the induction of MMP-1 in a UVA1-exposed condition. Results obtained with one of the main pro-inflammatory cytokines (IL-8) normalized by dermal fibroblasts number are presented in Figure 3d. On POMAOX treatment conditions, a decrease in IL-8 concentration was observed with a visible dose effect. At 0.0001% POMAOX, the level of IL-8 was comparable to that of the reference Vitamin C at 200 µM. Similarly, a significant decrease of all other cytokines’ concentration was observed at 0.0001% and 0.001% comparable for the latter to that of the reference, Vitamin C at 200 µM. The results obtained on all the different endpoints are summarized in Table 3 and Supplementary Data. Only TNF-α could not be detected, in all test conditions. This study showed that POMAOX at 0.001% presented a very strong photoprotective effect on all dermal damage endpoints including fibroblast disappearance and MMP1 release, as well as strong protection against UVA1-induced pro-inflammatory responses, with a decrease of pro-inflammatory cytokines IL-1α, IL-1ra, IL-6, IL-8, and GM-CSF. The effect of POMAOX was comparable to the effect of Vitamin C, confirming the strong potential of these new extracts to protect the skin from the deleterious impacts of UV exposure.

## 4. Discussion

Over the past decade, pomegranates have captured the attention of several scientific communities, fueled by a growing body of research, highlighting their remarkable cosmetic and therapeutic potential [11,25,26,27,28]. Pomegranate pericarps constitute approximately 40% of the whole fruit and are rich in ellagic acid derivatives such as ellagitannins, punicalagin, and punicalin. Remarkably, pomegranate juice is consumed throughout the world and constitutes punicalagins, the major ellagitannin that is responsible for more than 50% of the pomegranate juice’s potent antioxidant activity however, the aqueous medium is not appropriate to extract other key ellagitannins [29]. Therefore, our patented technology “POMAOX” aimed at optimizing the ellagitannin composition through a one-pot green extraction to constitute a composition containing 42% polyphenols chiefly comprising of ellagic acid, punicalins, and punicalagins and the subsequent investigation of this composition as a potent natural antioxidant (Table 4). Numerous studies have been reported on *Punica granatum*, one, in particular, discloses the strong protective benefit of the fruit juice, an extract and seed oil against UVB radiation-mediated damage and photoaging of human skin in a 3D full-thickness human reconstituted skin model [30]. Syed et al. reported the benefits of pomegranate fruit juice on normal human epidermal keratinocytes (NHEK) with significant protection against UVA-mediated activation of STAT3, AKT, and extracellular signal-regulated kinase (ERK1/2) pathways, leading to increased Ki-67 and proliferating cell nuclear antigen (PCNA) markers [11,29]. In addition, You-cheng et al. reported ellagic acid pretreatment markedly increased HaCaT cell viability and suppressed UVA-induced ROS generation and MDA formation [30,31]. Nevertheless, no study has been reported on the protection offered by a pomegranate pericarp extract against UVA-mediated oxidative stress and UVA1-induced dermal damage in a 3D human skin model. Our study on POMAOX demonstrated for the first time the antioxidant, anti-inflammatory, and protective properties against UV-induced MMP-1 production of a patented pomegranate extract.

To assess the antioxidant activity of POMAOX and build a correlation between the ellagitannin composition and its antioxidant activity, we investigated POMAOX for its electron-rich polyphenolic composition which are good electron donors to quench free radicals. The results from HORAC, SOAC, and NORAC showed moderate contributions to antioxidant activity with 8325 µg GAE/g, 220 ppm, and 1.0–3.0 ppm respectively, comparatively lower to that of the reference, vitamin C, except for NORAC values that were equivalent to vitamin C for POMAOX. Nitric oxide is an important intracellular messenger molecule and gets converted rapidly to NO_2_ under aerobic conditions. Prior et al., suggested that NO_2_ can be converted to peroxynitrite or nitric oxide and can react with superoxide anion to form peroxynitrite. Peroxynitrite oxidizes protein thiols forming disulfides and can nitrate tyrosyl groups in proteins to form nitrated proteins leading to either a loss or gain of enzymatic activity. Therefore, it can be envisaged that POMAOX could possibly protect most proteins and also a component of glutathione (GSH or GSSG), γ-Glu-Cys-Gly tripeptide [32] based on its strong NORAC values in the assay. In the past, studies revealed that a pomegranate fruit extract standardized with 30% punicalagin and 2.3% ellagic acid decreased hydrogen peroxide-induced ROS production by 1.36, 1.07, and 1.03-fold, respectively, in HaCaT cell cultures. Additionally, the peel extract also demonstrated significant free radical scavenging (81%) and lipid peroxidation (56%) protection [33]. Moreover, the fruit extract and its active compounds, punicalagin and ellagic acid were also reported to possess promising antioxidant effects by decreasing the level of hydrogen peroxide-induced apoptosis and downregulation of the levels of caspase-3 and caspase-7 in these cells [34]. Interestingly, ellagic acid was reported by Djedjibegovic et al., to have an ORAC value of 4.35 Trolox equivalent, two times lower than that of the flavonol, quercetin, and also lower than that of urolithin A (6.67 Trolox equivalent), and urolithin B (5.77 Trolox equivalent) describing ellagic acid to be a strong antioxidant from pomegranate [35]. It is generally accepted that radical oxygen quenching is a key requirement for an efficient antioxidant. However, the antioxidant capacity does not depend solely on the efficiency of quenching of the oxidizing radicals but also on the reactivity and the lifetime of the products of the quenching reaction. This is the first report attributing the antioxidant activity of a POMAOX extract against a battery of radical species to its phenolic composition, thereby suggesting its promising cosmetic potential in combating oxidative stress through ROS quenching.

Exposure to UVA increases the generation of reactive oxygen species (ROS) causing lipid peroxidation in cell membranes, which in turn depletes the levels of the endogenous antioxidant system leading to cell damage. This oxidative stress accelerates matrix metalloproteinases (MMPs) production, (especially MMP1), leading to collagen and elastin degradation in human skin, implicating sunlight as a major factor in photoaging and even skin cancer [31]. Dermal fibroblasts release MMP-1, following exposure to both UVB and UVA components of the solar spectrum, either through direct damage within the irradiated cells or by cytokine release and other soluble factors, produced in the skin in response to UV [36]. MMP-1 plays a critical role in the early stages of photoaging. As a key enzyme in the matrix metalloproteinase (MMP) family, MMP1 possesses the unique ability to break down collagen I and III, the main structural proteins of the skin’s extracellular matrix (ECM). By initiating the degradation of these collagen fibers, MMP-1 sets in motion a cascade of events that ultimately leads to ECM remodeling and breakdown, contributing significantly to the visible signs of aging caused by sun exposure [37,38]. Therefore, our investigation was focused on the protective effects of POMAOX on dermal fibroblasts, first comparing it to Vitamin E in a model of human dermal fibroblasts exposed to UVA radiation and measuring the release of 8-isoprostanes as a marker of oxidative stress targeting the membrane’s lipids. Fibroblasts pre-treated with POMAOX at 0.001% displayed a lower release of 8-iso-prostanes under UVA exposure, with protection against lipid peroxidation of 74%, while ellagic acid at 5 µM showed only 40% protection against lipid peroxidation. Interestingly, Khan et al. reported the peel extract of pomegranate at 0.2% *w*/*w* to reduce lipid peroxidation in a UVB-induced mouse model with increased levels of MDA, the byproduct of lipid peroxidation [39].

In the past, several investigations revealed that UV causes an inflammatory response in the human fibroblasts, resulting in both intrinsic and extrinsic aging [40]. It can directly or indirectly trigger several proinflammatory mediators, such as prostaglandin E2 (PGE2), cyclooxygenase-2 (COX-2), inducible nitric oxide synthase (iNOS), tumor necrosis factor-α (TNF-α), interleukin-1β (IL-1β) and interleukin-6 (IL-6) receptors [41]. Furthermore, UVA induces damage in skin fibroblasts and is reported to be associated not only with a decrease in mitochondrial function i.e., in oxygen consumption, mitochondrial membrane potential, and ATP content [42], reflecting a perturbation of energy metabolism but also an upregulation of the collagen degrading enzyme MMP-1 [43]. Pro-inflammatory cytokines, such as TNF-α, IL-6, and IL-1β, play an important role in the inflammatory response and also induce expression of MMP-1. In particular, MMP-1 and IL-8 are also associated with changes in fibroblast morphology as the latter acts as a chemotactic and activation factor for neutrophils and T lymphocytes [44]. Notably, these inflammation responses are also associated with skin fibroblast damage, which accelerates the photoaging process induced by UV irradiation. Our results indicate that POMAOX possibly suppresses the expression of MMP-1 and proinflammatory cytokines probably through the regulation of NF-κB and MAPK signaling pathways similar to pomegranate peel polyphenols (PPPs) reported earlier by Du et al., either by blocking LPS-induced phosphorylation, ubiquitination, and degradation of IκB [45]. Additionally, Rahman et al. and Behl et al. [46,47] showed that magnolol, a naturally occurring phenolic molecule possesses anti-inflammatory properties through TLR-4-mediated MAPK signaling, although not validated by us in this study. Besides the MAPK signaling pathway, the molecular mechanism of anti-inflammatory on PPPs is also associated with the NF-κB pathway. PPPs and their main components could inhibit NF-κB activation by blocking LPS-induced phosphorylation, ubiquitination, and degradation of IκB and, subsequently, could prevent p65 nuclear translocation [48,49].

We then investigated the protective effect of POMAOX on a full-thickness reconstructed skin model, T-skin^TM^, exposed to UVA1. The T-Skin™ is an in vitro reconstructed skin model that closely mirrors the structure of natural human skin. It consists of a dermal layer composed of human fibroblasts, upon which sits a multi-layered, well-differentiated epidermis derived from normal human keratinocytes. This standardized, commercially available model is based on established full-thickness skin models and offers a highly relevant platform for research [49,50,51]. This model serves as a powerful tool for evaluating the harmful impacts of UVA, allowing us to assess and quantify key mediators in exposed control and treated culture samples. Previous studies have demonstrated that UVA1 exposure in full-thickness reconstructed skin led to the generation of ROS in the dermal compartment, accompanied by alterations of fibroblast gene expression levels and increased secretion of MMPs and pro-inflammatory cytokines [5]. Oxidative stress is known to play a crucial role in MAP kinase-mediated signal transduction, which is responsible for UV-induced MMP-1 via the AP-1 pathway [21,22,41,52,53].

In this study, we were able to demonstrate the protective effect of the POMAOX extract at the molecular, cellular, and tissue levels. A strong photoprotective effect was observed both on the morphological structure, UVA1-induced MMP-1 release, and pro-inflammatory responses (IL-1α, IL-1ra, IL-6, IL-8, and GM-CSF) after UVA1 exposure. Recent studies by Oh et al. reported that a lignan polyphenol, (±)-syringaresinol at 1, 5, and 20 μM suppressed MMP-1 release and enhanced collagen production in UVA irradiated HaCaT keratinocytes and human dermal fibroblasts [51]. The authors also demonstrated the potential of the lignan polyphenol to inhibit UVA-induced production of inflammatory cytokines, TNF-α, COX-2, IL-1β, and IL-6 in human HaCaT keratinocytes and dermal fibroblasts in a dose effective manner [47]. The study offered insights into how polyphenols inhibit MMP-1 production via blocking MAPK-cascade-regulated AP-1 transcriptional activity which is a result of UVA-induced inflammatory response in the skin [5,54].

Reduction in collagen density and increased MMP-1 secretion on UVA exposure in both ex-vivo human skin and reconstructed skin has been well documented while cosmetic ingredients and sunscreen formulas have been shown to demonstrate anti-photoaging efficacy on the UV-associated biological markers [55]. The impact of UVA1-induced injuries such as the generation of reactive oxygen species and thymine dimers DNA damage generally accumulate preferentially in dermal fibroblasts and basal keratinocytes [56]. A full genome transcriptomic study showed clear UVA1 molecular signatures with the expression of 461 and 480 genes in epidermal keratinocytes and dermal fibroblasts, suggesting the extent of modulated genes related to innate immunity and inflammation [5]. Further, exposure to UVA can also lead to reactive oxygen species (ROS) and reactive nitrogen species (RNS) from inflammatory and epithelial cells, and thereby result in the formation of oxidative and nitrative DNA lesions, such as 8-oxo-7,8-dihydro-2′-deoxyguanosine (8-oxodG) and 8-nitroguanine, important markers for oxidative stress, mutagenesis, and carcinogenesis [5,55]. Concurringly, Afaq et al. reported the protective effect of pomegranate juice, extract, and oil at 5–10 µg/mL on UVB-mediated damage in a 3D full-thickness human reconstituted skin model by inhibiting the formation of cyclobutane pyrimidine dimers (CPDs) and 8-dihydro-2′ -deoxyguanosine (8-OHdG), which represent important biomarkers of DNA damage induced by UVB [29]. Therefore, it is plausible that our POMAOX extract could be involved in inhibiting 8-oxodG being an important marker in UVA-induced oxidative stress, although not investigated in this study and is planned to be taken up in the near future.

## 5. Conclusions

In conclusion, our pomegranate-derived extract, POMAOX demonstrated significant protective effects against UVA-induced damage in human skin, including protection against oxidative stress, inhibition of the collagen-degrading enzyme MMP-1 release, and inhibition of the production of proinflammatory cytokine mediators. These findings suggest that POMAOX could offer enhanced protection against the detrimental effects of UV exposure in the dermis leading to photoaging signs and could thereby address the growing consumer demand for strong photoprotection with skincare benefits. Given the promising protective effects observed for POMAOX on human skin, future in vivo studies are warranted to investigate its efficacy and elucidate its mechanisms of action, paving the way for the development of POMAOX-based cosmeceuticals.

## 6. Patents

WIPO (PCT) WO2021064034A1: A *Punica granatum* extract and its cosmetic uses. International patent classification A61K 8/9789 (2017.01), A61Q 19/08 (2006.01) and A61Q 17/04 (2006.01), International application number: PCT/EP2020/0773, Filling date: 30 September 2020. Steve Thomas Pannakal and Arun Duraisamy.

## Figures and Tables

**Table 1:** Antioxidant capacity (ORAC, SOAC, SORAC, NORAC, and HORAC) of POMAOX and Vitamin C.

Test Compounds	Antioxidant Capacity				
	ORAC μg (TE/g)	SOAC at 50% Inhibition (ppm)	SORAC (U/mg)	NORAC (ppm)	HORAC (µg/g)
Radical	ROO.	^1^O_2_.	-O_2_.	-NOO.	HO.
Reference	TROLOX	Vitamin E	SOD Enzyme	Vitamin C	Gallic Acid
POMAOX	NA	220	500	1–3	8325
Vitamin C	1.05	0.66	7.99	1.0	0.02

NA: Not active in the assay.

**Table 2:** MTT assay, Solubility and Cytotoxicity of POMAOX.

Test Compound	MTT and Treatment Concentrations	Vehicle	Solubility	Cytotoxicity (Viability + Morphology)
POMAOX	0.0001–0.001–0.005%	DMSO 1/1000	Good	At 0.005%

**Table 3:** Photoprotective effects summary of POMAOX in the UVA1 photoprotection test on T-Skin™ model. All the results summarized were obtained after UVA1 exposure at 35 J/cm^2^. Results were normalized by dermal fibroblast counting and expressed as a percentage of the UVA1-exposed control (DMSO 0.1% for POMAOX, untreated for Vitamin C) DMSO 0.1% UVA1-exposed control showed similar results to untreated, UVA1-exposed control. The values represent mean +/− SD of three independent batches of tissues.

Endpoint	Untreated	Vitamin C 200 μM	POMAOX (*PUNICA GRANATUM EXTRACT*)	
			0.0001%	0.001%
**FHN**	**100** +/− 49	**379** +/− 157	**161** +/− 40	**260** +/− 63
***MMP-1***	**100** +/− 41	**26** +/− 12	**67** +/− 23	**38** +/− 14
***IL-1a***	**100** +/− 55	**16** +/− 7	**26** +/− 10	**16** +/− 9
***IL-1ra***	**100** +/− 58	**21** +/− 6	**27** +/− 11	**21** +/− 13
***IL-6***	**100** +/− 28	**38** +/− 27	**77** +/− 36	**35** +/− 18
***IL-8***	**100** +/− 36	**26** +/− 14	**67** +/− 30	**29** +/− 16
***GM-CSF***	**100** +/− 51	**19** +/− 14	**28** +/− 18	**24** +/− 10

**Table 4:** POMAOX Analytical composition.

Parameters	Values in % *w*/*w*	Method
Punicalagins	14.44%	HPLC-UV quantification with Reference standards
Ellagic acid	14.12%	
Punicalins	3.28%	
Total polyphenols (punicalins, punicalagins and ellagic acid, gallic acid, epi-catechin gallate, gallagic acid, ellagic acid hexoside/glycoside)	42%	Folin-Ciocalteu method
Total fats	5.6%	Acid hydrolysis method
Total protein	0.73%	Kjeldahl Method
Total sugars	19.1%	Phenol-sulfuric acid method
Total saponins	7.6%	Gravimetric method
Moisture	6.5%	Thermogravimetric approach (Loss on Drying)

## Data Availability

Data is contained within the article or Supplementary Materials.

## Supplementary Materials

The following supporting information can be downloaded at: https://www.mdpi.com/article/10.3390/antiox14030301/s1.

## References

[1] Sander C.S., Chang H., Hamm F., Elsner P., Thiele J.J. (2004). Role of oxidative stress and the antioxidant network in cutaneous carcinogenesis. Int. J. Dermatol..

[2] Bernerd F., Passeron T., Castiel I., Marionnet C. (2022). The Damaging Effects of Long UVA (UVA1) Rays: A Major Challenge to Preserve Skin Health and Integrity. Int. J. Mol. Sci..

[3] Skarupova D., Vostalova J., Rajnochova Svobodova A. (2020). Ultraviolet A protective potential of plant extracts and phytochemicals. Biomed. Pap..

[4] Amano S. (2016). Characterization and mechanisms of photoageing-related changes in skin. Damages of basement membrane and dermal structures. Exp. Dermatol..

[5] Marionnet C., Pierrard C., Golebiewski C., Bernerd F. (2014). Diversity of biological effects induced by longwave UVA rays (UVA1) in reconstructed skin. PLoS ONE.

[6] Briganti S., Picardo M. (2003). Antioxidant activity, lipid peroxidation and skin diseases. What’s new. J. Eur. Acad. Dermatol. Venereol..

[7] Holland D., Hatib K., Bar-Ya’akov I. (2009). Pomegranate: Botany, horticulture, breeding. Horticul. Rev..

[8] Dar M., Shrestha R., Cochard R. (2012). Plant resource utilization by local inhabitants around Machiara National Park, District. J. Food Agric. Environ..

[9] Montes de Oca M.K., Pearlman R.L., McClees S.F., Strickland R., Afaq F. (2017). Phytochemicals for the Prevention of Photocarcinogenesis. Photochem. Photobiol..

[10] Kang S.J., Choi B.R., Kim S.H., Yi H.Y., Park H.R., Park S.J., Song C.H., Park J.H., Lee Y.J., Kwang S. (2015). Inhibitory effects of pomegranate concentrated solution on the activities of hyaluronidase, tyrosinase, and metalloproteinase. J. Cosmet. Sci..

[11] Syed D.N., Malik A., Hadi N., Sarfaraz S., Afaq F., Mukhtar H. (2006). Photochemopreventive effect of pomegranate fruit extract on UVA-mediated activation of cellular pathways in normal human epidermal keratinocytes. Photochem. Photobiol..

[12] Matsui M.S. (2016). The Role of Topical Antioxidants in Photoprotection.

[13] Clementi M.E., Sampaolese B., Sciandra F., Tringali G. (2020). Punicalagin Protects Human Retinal Pigment Epithelium Cells from Ultraviolet Radiation-Induced Oxidative Damage by Activating Nrf2/HO-1 Signaling Pathway and Reducing Apoptosis. Antioxidants.

[14] Scarpin M.S., Kawakami C.M., Pereira K.C., Benevenuto C.G., Gaspar L.R. (2021). Effects of UV-filter photostabilizers in the photostability and phototoxicity of vitamin A palmitate combined with avobenzone and octyl methoxycinnamate. Photochem. Photobiol..

[15] Ou B., Chang T., Huang D., Prior R.L. (2013). Determination of total antioxidant capacity by oxygen radical absorbance capacity (ORAC) using fluorescein as the fluorescence probe: First action. J. AOAC Intern..

[16] Zhang L., Huang D., Kondo M., Fan E., Ji H., Kou Y., Ou B. (2009). Novel high-throughput assay for antioxidant capacity against superoxide anion. J. Agric. Food Chem..

[17] Ou B., Hampsh-woodill M., Flanagan J., Deemer E.K., Prior R.L., Huang D. (2002). Novel fluorometric assay for hydroxyl radical prevention capacity using fluorescein as the probe. J. Agric. Food Chem..

[18] Ou B., Zhang L., Kondo M., Ji H., Kou Y. (2012). Method for Assaying the Antioxidant Capacity of a Skin Care Product.

[19] Kazuo M. (2019). Antioxidant Activity of Foods: Development of Singlet Oxygen Absorption Capacity (SOAC) Assay Method. J. Nutr. Sci. Vitaminol..

[20] Andrés C.M.C., Lastra J.M.P., Juan C.A., Plou F.J., Pérez-Lebeña E. (2023). Chemical Insights into Oxidative and Nitrative Modifications of DNA. Int. J. Mol. Sci..

[21] Lelièvre D., Canivet F., Thillou F., Tricaud C., Le Floc’h C., Bernerd F. (2024). Use of reconstructed skin model to assess the photoprotection afforded by three sunscreen products having different SPF values against DNA lesions and cellular alterations. J. Photochem. Photobiol..

[22] Bataillon M., Lelievre D., Chapuis A. (2019). Characterization of a New Reconstructed Full Thickness Skin Model, T-Skin™, and its Application for Investigations of Anti-Aging Compounds. Int. J. Mol. Sci..

[23] Fisher G.J., Wang Z.Q., Datta S.C., Varani J., Kang S., Voorhees J.J. (1997). Pathophysiology of premature skin aging induced by ultraviolet light. N. Engl. J. Med..

[24] Marionnet C., Nouveau S., Hourblin V., Pillai K., Manco M., Bastien P., Tran C., Tricaud C., de Lacharrière O., Bernerd F. (2017). UVA1-Induced Skin Darkening Is Associated with Molecular Changes Even in Highly Pigmented Skin Individuals. J. Investig. Dermatol..

[25] Chen B., Tuuli M.G., Longtine M.S., Shin J.S., Lawrence R., Inder T., Michael Nelson D. (2012). Pomegranate juice and punicalagin attenuate oxidative stress and apoptosis in human placenta and in human placental trophoblasts. Am. J. Physiol. Endocrinol. Metab..

[26] Zeghad N., Ahmed E., Khan M.Z., Belkhiri A. (2023). Exploring the potential use of pomegranate (*Punica granatum* L.) and prickly pear (*Opuntia ficus indica* L.) peels as sources of cosmeceutical sunscreen agent for their antioxidant and photoprotective properties. Pharm. Sci. Asia.

[27] Li L., Chong L., Huang T., Ma Y., Li Y., Ding H. (2023). Natural products and extracts from plants as natural UV filters for sunscreens: A review. Anim. Model Exp. Med..

[28] Shahkoomahally S., Shin D., Habibi F., Kim J., Sarkhosh A. (2023). Profiling phenolic compounds in juice and peel of fourteen pomegranate (*Punica granatum* L.) varieties grown in Florida, USA. Food Chem. Adv..

[29] Afaq F., Abu Zaid M., Khan N., Dreher M., Mukhtar H. (2009). Protective effect of pomegranate-derived products on UVB-mediated damage in human reconstituted skin. Exp. Dermatol..

[30] Hseu Y.C., Chou C.W., Kumar K.J.S., Fu K.T., Wang H.M., Hsu L.S., Kuo Y.H., Wu C.R., Chen S.C., Yang H.L. (2012). Ellagic acid protects human keratinocyte (HaCaT) cells against UVA-induced oxidative stress and apoptosis through the upregulation of the HO-1 and Nrf-2 antioxidant genes. Food Chem. Toxicol..

[31] Dong K.K., Damaghi N., Picart S.D. (2008). UV-induced DNA damage initiates release of MMP-1 in human skin. Exp. Dermat..

[32] Prior R.L., Sintara M., Chang T. (2016). Multi-radical (ORAC MR5) antioxidant capacity of selected berries and effects of food processing. J. Berry Res..

[33] Singh B., Singh J.P., Kaur A., Singh N. (2018). Phenolic compounds as beneficial phytochemicals in pomegranate (*Punica granatum* L.) peel: A review. Food Chem..

[34] Liu C., Guo H., DaSilva N.A., Li D., Zhang K., Wan Y., Gao X.-H., Chen H.-D., Seeram N.P., Ma H. (2019). Pomegranate (*Punica granatum*) Phenolics Ameliorate Hydrogen Peroxide-Induced Oxidative Stress and Cytotoxicity in Human Keratinocytes. J. Funct. Foods.

[35] Djedjibegovic J., Marjanovic A., Panieri E., Saso L. (2020). Ellagic Acid-Derived Urolithins as Modulators of Oxidative Stress. Oxid. Med. Cell Longev..

[36] Kossodo S., Wong W.R., Simon G., Kochevar I.E. (2004). Effects of UVR and UVR-induced cytokines on production of extracellular matrix proteins and proteases by dermal fibroblasts cultured in collagen gels. Photochem. Photobiol..

[37] Martin R., Pierrard C., Lejeune F., Hilaire P., Breton L., Bernerd F. (2008). Photoprotective effect of a water-soluble extract of Rosmarinus officinalis L. against UV-induced matrix metalloproteinase-1 in human dermal fibroblasts and reconstructed skin. Eur. J. Dermatol..

[38] Fagot D., Asselineau D., Bernerd F. (2004). Matrix metalloproteinase-1 production observed after solar-simulated radiation exposure is assumed by dermal fibroblasts but involves a paracrine activation through epidermal keratinocytes. Photochem. Photobiol..

[39] Khan N., Syed D.N., Pal H.C., Mukhtar H., Afaq F. (2012). Pomegranate fruit extract inhibits UVB-induced inflammation and proliferation by modulating NF-κB and MAPK signaling pathways in mouse skin. Photochem. Photobiol..

[40] Franceschi C., Campisi J. (2014). Chronic Inflammation (Inflammaging) and Its Potential Contribution to Age-Associated Diseases. J. Gerontol. Ser. A Biomed. Sci. Med. Sci..

[41] Bickers D.R., Athar M. (2006). Oxidative Stress in the Pathogenesis of Skin Disease. J. Investig. Dermatol..

[42] Yamawaki Y., Mizutani T., Okano Y., Masaki H. (2019). The impact of carbonylated proteins on the skin and potential agents to block their effects. Exp. Dermatol..

[43] Berneburg M., Gremmel T., Kürten V., Schroeder P., Hertel I., Von Mikecz A., Wild S., Chen M., Declercq L., Matsui M. (2005). Creatine supplementation normalizes mutagenesis of mitochondrial DNA as well as functional consequences. J. Investig. Dermatol..

[44] Berneburg M., Plettenberg H., Krutmann J. (2000). Photoaging of human skin. Photodermatol. Photoimmunol. Photomed..

[45] Du L., Li J., Zhang X., Wang L., Zhang W., Yang M., Hou C. (2019). Pomegranate peel polyphenols inhibits inflammation in LPS-induced RAW264.7 macrophages via the suppression of TLR4/NF-κB pathway activation. Food Nutr. Res..

[46] Rahman M.M., Islam M.R., Akash S., Hossain M.E., Tumpa A.A., Abrar A.G.M., Ahmed L., Rauf A., Khalil A.A., Al Abdul monem W. (2023). Pomegranate-specific natural compounds as onco-preventive and onco-therapeutic compounds: Comparison with conventional drugs acting on the same molecular mechanisms. Heliyon.

[47] Behl T., Upadhyay T., Singh S., Chigurupati S., Alsubayiel A.M., Mani V., Vargas-De-La-Cruz C., Uivarosan D., Bustea C., Sava C. (2021). Polyphenols Targeting MAPK Mediated Oxidative Stress and Inflammation in Rheumatoid Arthritis. Molecules.

[48] Asselineau D., Bernard B.A., Bailly C., Darmon M. (1985). Epidermal morphogenesis and induction of 67kD keratin polypeptide by culture at the liquid-air interface. Exp. Cell Res..

[49] Bernerd F., Asselineau D. (1997). Successive alteration and recovery of epidermal differentiation and morphogenesis after specific UVB-damages in skin reconstructed in vitro. Dev. Biol..

[50] Oh J.H., Joo Y.H., Karadeniz F., Ko J., Kong C.S. (2020). Syringaresinol Inhibits UVA-Induced MMP-1 Expression by Suppression of MAPK/AP-1 Signaling in HaCaT Keratinocytes and Human Dermal Fibroblasts. Int. J. Mol. Sci..

[51] de Jager T.L., Cockrell A.E., Du Plessis S.S. (2017). Ultraviolet light induced generation of reactive oxygen species. Adv. Exp. Med. Bio..

[52] Watanabe H., Shimizu T., Nishihira J., Abe R., Nakayama T., Taniguchi M., Sabe H., Ishibashi T., Shimizu H. (2004). Ultraviolet A-induced production of matrix metalloproteinase-1 is mediated by macrophage migration inhibitory factor (MIF) in human dermal fibroblasts. J. Biol. Chem..

[53] Wenk J., Brenneisen P., Wlaschek M., Poswig A., Briviba K., Oberley T.D., Scharffetter-Kochanek K. (1999). Stable overexpression of manganese superoxide dismutase in mitochondria identifies hydrogen peroxide as a major oxidant in the AP-1-mediated induction of matrix degrading metalloprotease-1. J. Biol. Chem..

[54] Liu Y., Liu J., Dai H., Wang R., Hsiao A., Wang W., Betts R.J., Marionnet C., Bernerd F., Qiu J. (2022). Photo-aging evaluation—In vitro biological endpoints combined with collagen density assessment with multi-photon microscopy. J. Dermatol. Sci..

[55] Cadet J., Douki T. (2011). Oxidatively generated damage to DNA by UVA radiation in cells and human skin. J. Investig. Dermatol..

[56] Lee C.H., Wu S.B., Hong C.H., Yu H.S., Wei Y.H. (2013). Molecular Mechanisms of UV-Induced Apoptosis and Its Effects on Skin Residential Cells: The Implication in UV-Based Phototherapy. Int. J. Mol. Sci..

