# Supplementary figures and images for: A Proprietary Punica granatum pericarp Extract, Its Antioxidant Properties Using Multi-Radical Assays and Protection Against UVA-Induced Damages in a Reconstructed Human Skin Model
Source: Antioxidants (Basel). 2025 Feb 28;14(3):301. doi: 10.3390/antiox14030301 (PMC11939174; doi:10.3390/antiox14030301)

# Supplementary data

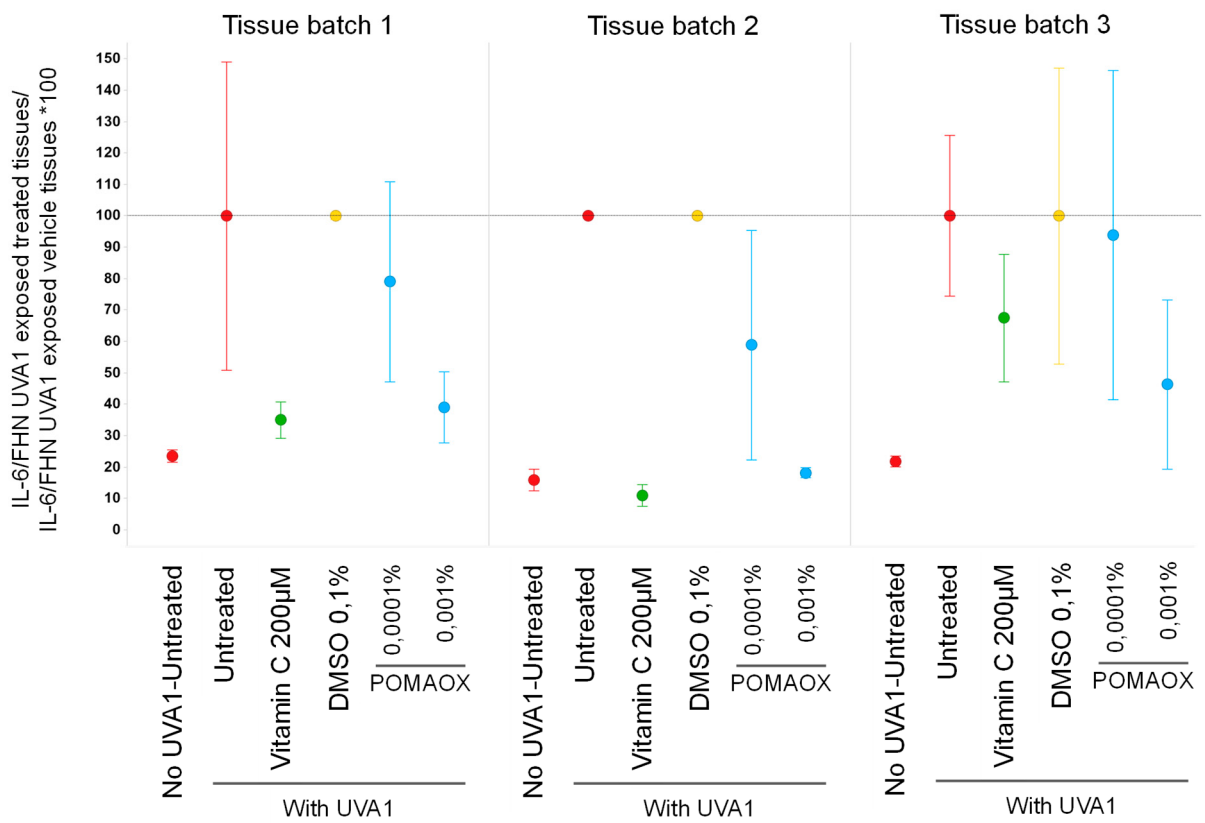

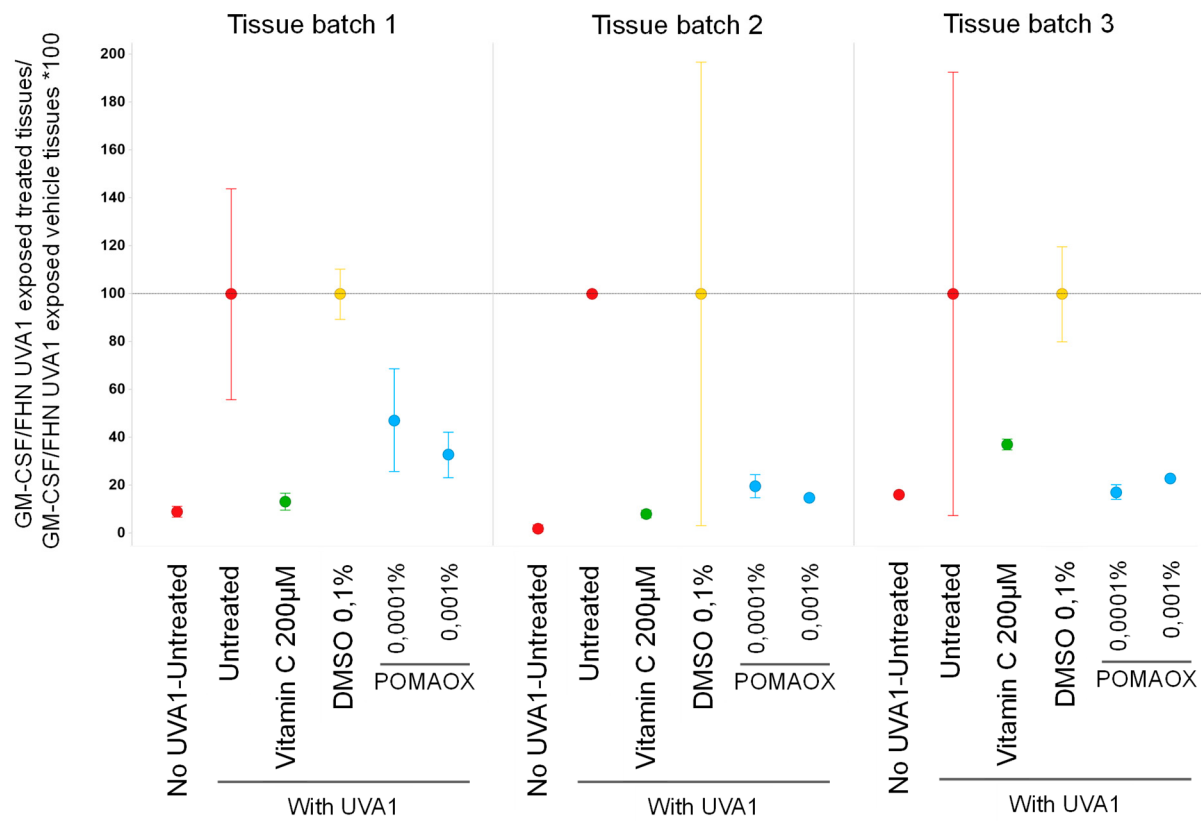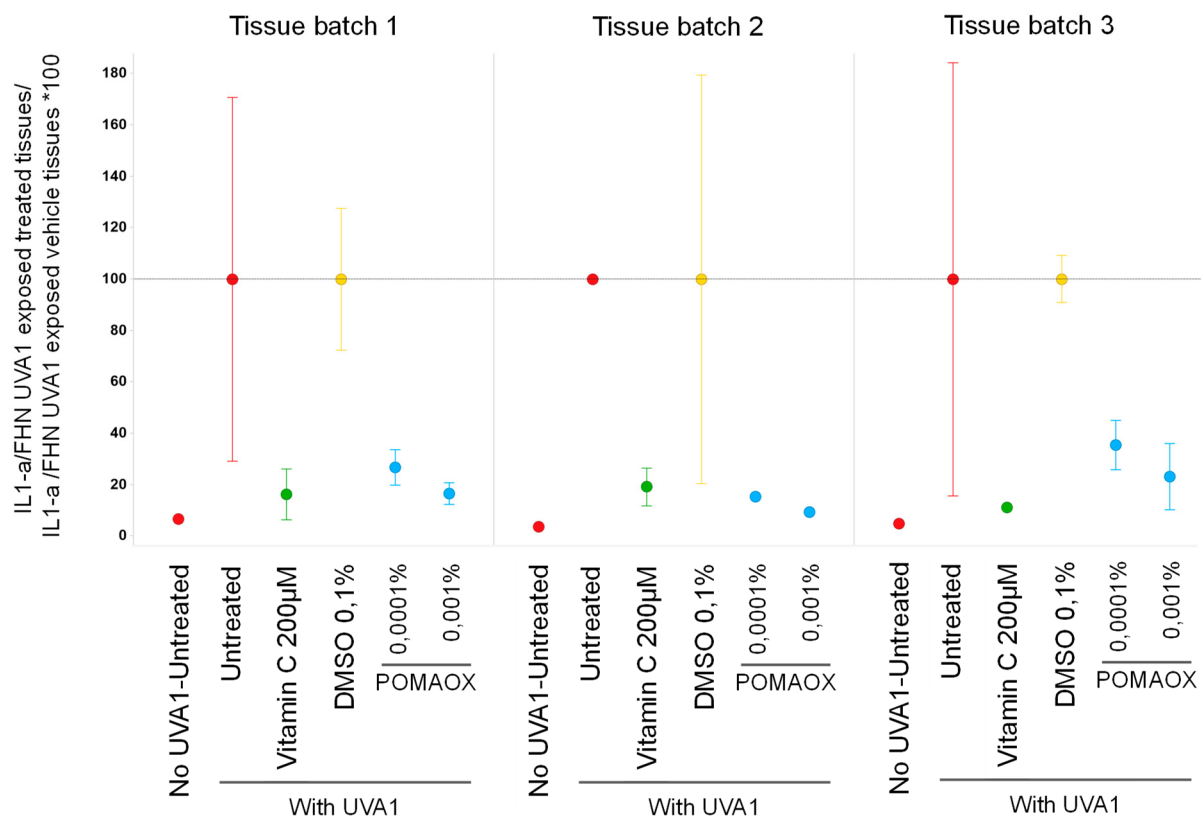

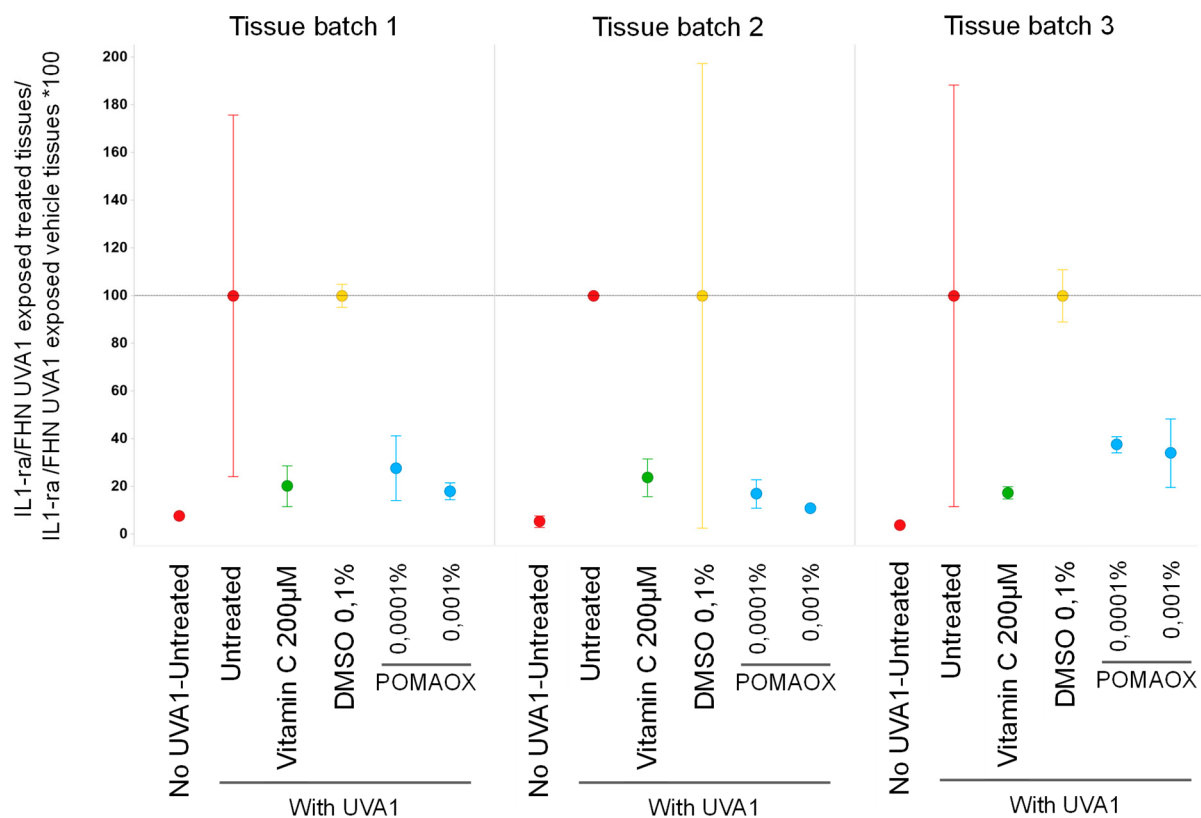

Supplement: Supplementary file 1 [file antioxidants-14-00301-s001.zip › antioxidants-3424563-supplementary.pdf]
